# A multi-level spatial analysis of clinical malaria and subclinical *Plasmodium* infections in Pailin Province, Cambodia

**DOI:** 10.1016/j.heliyon.2017.e00447

**Published:** 2017-11-20

**Authors:** Daniel M. Parker, Rupam Tripura, Thomas J. Peto, Richard J. Maude, Chea Nguon, Jeremy Chalk, Pasathorn Sirithiranont, Mallika Imwong, Lorenz von Seidlein, Nicholas J. White, Arjen M. Dondorp

**Affiliations:** a Shoklo Malaria Research Unit, Mae Sot, Thailand; b Mahidol Oxford Tropical Medicine Research Unit, Faculty of Tropical Medicine, Mahidol University, Bangkok, Thailand; c Center of Tropical Medicine and Travel Medicine, Department of Infectious Diseases, Division of Internal Medicine, Academic Medical Center, University of Amsterdam, Amsterdam, The Netherlands; d Centre for Tropical Medicine and Global Health, Nuffield Department of Clinical Medicine, University of Oxford, Oxford, UK; e National Center for Parasitology, Entomology and Malaria Control, Phnom Penh, Cambodia; f Department of Epidemiology, Harvard T. H. Chan School of Public Health, Harvard University, Boston, USA; g Department of Molecular Tropical Medicine and Genetics, Faculty of Tropical Medicine, Mahidol University, Bangkok, Thailand

**Keywords:** Infectious disease, Public health, Geography, Information science

## Abstract

**Background:**

The malaria burden is decreasing throughout the Greater Mekong Subregion, however transmission persists in some areas. Human movement, subclinical infections and complicated transmission patterns contribute to the persistence of malaria. This research describes the micro-geographical epidemiology of both clinical malaria and subclinical *Plasmodium* infections in three villages in Western Cambodia.

**Methods:**

Three villages in Western Cambodia were selected for the study based on high reported *Plasmodium falciparum* incidence. A census was conducted at the beginning of the study, including demographic information and travel history. The total population was 1766. Cross-sectional surveys were conducted every three months from June 2013 to June 2014. *Plasmodium* infections were detected using an ultra-sensitive, high-volume, quantitative polymerase chain reaction (uPCR) technique. Clinical episodes were recorded by village health workers. The geographic coordinates (latitude and longitude) were collected for all houses and all participants were linked to their respective houses using a demographic surveillance system. Written informed consent was obtained from all participants.

**Results:**

Most clinical episodes and subclinical infections occurred within a single study village. Clinical *Plasmodium vivax* episodes clustered spatially in each village but only lasted for a month. In one study village subclinical infections clustered in geographic proximity to clusters of clinical episodes. The largest risk factor for clinical *P. falciparum* episodes was living in a house where another clinical *P. falciparum* episode occurred (model adjusted odds ratio (AOR): 6.9; CI: 2.3–19. 8). Subclinical infections of both *P. vivax* and *P. falciparum* were associated with clinical episodes of the same species (AOR: 5.8; CI: 1.5–19.7 for *P. falciparum* and AOR: 14.6; CI: 8.6–25.2 for *P. vivax*) and self-reported overnight visits to forested areas (AOR = 3.8; CI: 1.8–7. 7 for *P. falciparum* and AOR = 2.9; CI: 1.7–4.8 for *P. vivax*).

**Discussion:**

Spatial clustering within the villages was transient, making the prediction of spatial clusters difficult. Interventions that are dependent on predicting spatial clusters (such as reactive case detection) would only have detected a small proportion of cases unless the entire village was screened within a limited time frame and with a highly sensitive diagnostic test. Subclinical infections may be acquired outside of the village (particularly in forested areas) and may play an important role in transmission.

## 1 Introduction

In much of the Greater Mekong Subregion (GMS) malaria case numbers have been decreasing and nations are moving from malaria control to elimination. Village-based treatment of malaria is central to elimination strategies [1], however, in many parts of the GMS there is no functioning village health worker network, especially in areas near international borders [2]. Interventions such as insecticide treated bed nets (LLITNs) are less effective in the GMS than in Africa, since most of the important *Anopheles* vectors feed outdoors and during dusk and dawn when few people are sleeping [3].

Within the remaining malaria endemic areas in the GMS, malaria risk is heterogeneously distributed and the complete interruption of malaria transmission has thus far been elusive. In many parts of the GMS malaria persists in clusters along international borders as a result of a complex combination of political, environmental, and socio-economic factors that are not completely understood. Migration of both mosquitoes and humans can lead to the reintroduction of parasites to regions that might otherwise be malaria free [4]. A high prevalence of subclinical and submicroscopic *Plasmodium* infections, even in low transmission areas such as the GMS, undermines elimination efforts as these infections contribute to transmission and are unlikely to be detected and treated through conventional control activities [5, 6, 7].

Current elimination efforts are focused on *Plasmodium falciparum*. *Plasmodium vivax* presents particular problems to control and elimination because liver hypnozoites cause periodic relapses. The situation is compounded by safety concerns regarding the two-week treatment with primaquine required for the radical cure of hypnozoites [8, 9, 10].

Micro-geographical analyses can inform how, when, and where clustering occurs and how it relates to transmission patterns [11]. Clustering can occur at multiple levels: within hosts (individuals with repeated infections over time) and within populations (groups of hosts that share exposure sites such as houses, villages, or work environments). Major control and elimination strategies (such as targeted mass treatment with or without screening, reactive case detection, and focused screening and treatment) rely on being able to identify space-time clusters of malaria. This clustering can give information about transmission patterns. For example, if transmission occurs within villages then cases tend to cluster in particular groups of houses [12]. Conversely, if transmission occurs outside of the village then the disease tends to cluster in individuals who are exposed to transmission sites [13]. An understanding of local clustering patterns can be used to help design or select appropriate interventions.

The main goals of this research were to investigate potential associations between 1) subclinical *Plasmodium* infections and clinical malaria episodes, 2) travelling to or spending the night in the forest and clinical episodes or subclinical *Plasmodium* infections, and 3) spatial and spatio-temporal clustering of clinical episodes or subclinical *Plasmodium* infections within villages in Pailin Province, Cambodia.

## 2 Methods

### 2.1 Study sites

**Fig. 1:**
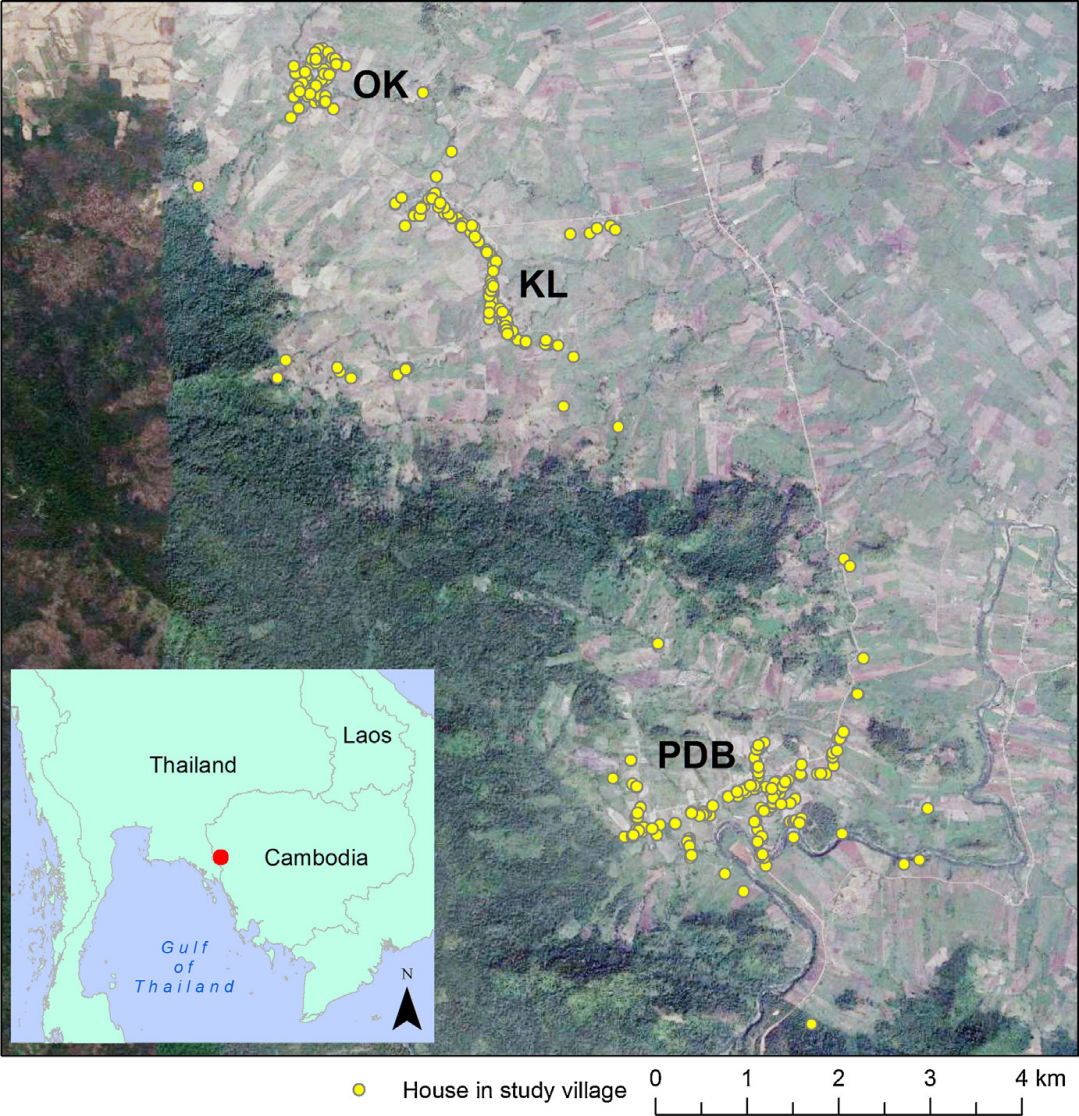
Map of study villages.

The study area consisted of three villages in Pailin Province, Cambodia, near the international border with Thailand (Fig. 1). In 2004 Pailin reported some of the highest annual parasite incidences (API) in Cambodia, with API above 160/per 1000 people per year for both *P. falciparum* and *P. vivax*. By 2013 this incidence had fallen to around 10/1000 for *P. falciparum* and 20/1000 for *P. vivax*. The areas of highest transmission were no longer in the west of Cambodia but were now in the north and north east [14]. The dramatic decline of malaria in western Cambodia may be attributable at least in part to the increased malaria control efforts following the detection of artemisinin resistant *P. falciparum* [15] with an important scale-up of the village malaria worker programme and improved coverage of insecticide treated bednets [16, 17]. Deforestation may also play a role [13]. Despite the fall in clinical incidence, a reservoir of subclinical malaria remains and is an important target for accelerated elimination strategies [5, 6, 7, 18].

The study villages were selected based on *P. falciparum* incidence in 2012 and are adjacent to each other, covering an area approximately ten km long and four km wide.

### 2.2 Data collection

Detailed demographic and geographic surveys were conducted beginning in June 2013 and ending in December of 2014. During the study period, first-line treatment for *P. falciparum* and for *P. vivax* was dihydroartemisinin-piperaquine. Primaquine was not used for *P. vivax* due to a lack of availability of G6PD testing. In addition radical cure of *P. vivax* with primaquine was not recommended in the national treatment guidelines at the time of the study. Individuals who agreed to participate in the study had their age and sex recorded and were given an identification code. The geographic coordinates (latitude and longitude) for all houses in the study villages were recorded and each house was assigned an identification code. All participating individuals were linked through a demographic surveillance system to their respective houses. All participants provided written informed consent.

Mass blood screenings were conducted in the villages every three months from June 2013 to June 2014, (M0–M12 [7]). During the cross-sectional surveys a mobile clinic was established in a central location within each village, usually in the village school. Village volunteers were trained to assist in the surveys and they went house-to-house to inform and invite all residents to participate. Surveys took place over several days and were usually followed up one week later in order to survey participants who were absent during the survey. Within each survey, house members attended as groups and passed through a series of desks where they provided informed consent, they were registered, weighed, measured, had their tympanic temperature taken, completed a short survey form, provided a venous blood sample and received a consultation with a nurse if necessary. During the screenings, 3 mL of blood was collected from each adult participant (500 uL from children six months to five years old), any malaria symptoms (e.g. fever, chills) were noted, and the blood was analyzed using an ultra-sensitive quantitative polymerase chain reaction method (uPCR), with a lower level of detection of 22 parasites per mL (described in detail in [5]).

Patients without clinical symptoms who had *Plasmodium* infection detected by uPCR are herein referred to as having “subclinical” infections. Anyone with a temperature of 37.5 °C or above had an rapid diagnostic test (RDT) performed by the village malaria worker and, if positive for malaria, was treated according to national guidelines.

Each village had a village malaria worker (VMW), which is the primary care provider for patients with febrile illness. The VMW provided diagnosis and treatment for symptomatic malaria cases. Patients who attended the VMW were recorded in a log book and matched to their study identification number. Patients were tested for malaria using a RDT (CareStart™ Malaria Pf/Pv Combo test) and treated as per national guidelines if found infected. Patient outcomes were recorded and for the present analysis were considered “clinical” episodes of *P. falciparum* or *P. vivax* malaria beginning in June 2013 and running through December 2014 (M0–M18). Both clinical episodes and subclinical infections were then linked to houses of residence for analysis.

Brief travel history and bednet usage interviews were also conducted during each of the three-monthly cross-sectional surveys. Participants were asked if they had travelled to another location or spent the night in the forest within the last two weeks.

The original census population was treated as a cohort for this analysis. Some individuals moved into the villages during the study period but were not included in this analysis as their data do not account for their previous exposures and environments. Participants who moved out of the cohort and villages during the study period [7] were included in this analysis.

### 2.3 Analysis

The outcome variables for these analyses were clinical episodes (of *P. falciparum* or *P. vivax*, separately) and subclinical infections (also of *P. falciparum* or *P. vivax* separately). Both clinical malaria episodes and subclinical infections detected by uPCR during surveys can occur multiple times within individual participants and all episodes were included in the analysis. It was not possible to determine whether or not recurrent malaria infections in individuals were new infections or recrudescences, as the genetic material was typically insufficient for genotyping. Also, relapse infections in *P. vivax* cannot be distinguished from recrudescent infection. Therefore, individuals who had either a confirmed clinical episode or uPCR-confirmed subclinical infection with malaria (for *P. falciparum* and *P. vivax* separately) were coded with a “1” regardless of their number of infections. Participants who never presented at a clinic with symptomatic malaria and who never had malaria parasites detected by uPCR during the study period were coded as “0”. A quantitative variable was included to account for the number of screenings attended by individuals, with some individuals only attending 1 screening and others attending all 5.

In order to test for house-level clustering of infections, two house-level covariates were also included to indicate whether or not there were secondary infections within the house: one indicating secondary clinical episodes and one indicating secondary uPCR detected infections. This covariate excluded the index case.

**Fig. 2:**
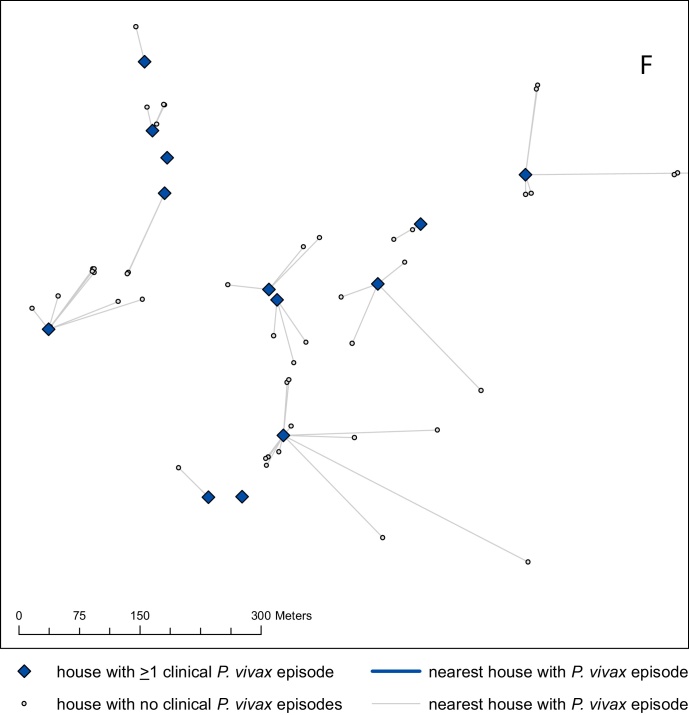
Figure indicating distance to the nearest neighbour with a clinical episode for houses with a clinical episode (red) and without a clinical episode (grey).

Categorical predictor variables were created for participant sex and age, with age being aggregated into groups based on work behaviors of villagers (age groups 0–9; 10–45; and 46 plus). Two predictor categorical variables related to human movement patterns were also created, one based on the participant reporting spending the night in the forest (1 = yes; 0 = no) and the other based on spending the night away from the village (1 = yes; 0 = no). Participants were asked whether or not they had spent 1 or more nights in the forest or traveling away from the village within the previous two weeks. A categorical variable was created for self-reported bednet use (1 = reported having regularly used bednets; 0 = reported not regularly using bednets). A continuous spatial variable that measured the distance from a house to the nearest house with a clinical infection (for clinical *P. falciparum* and clinical *P. vivax*) was generated (Fig. 2). A categorical variable for village was included both to account for unmeasured village-specific factors and clustering at the village level.

### 2.4 Exploratory analysis

Exploratory analyses investigated outcome and predictor variables with regard to outcome variables as well as the timing between episodes that occurred within house and within individuals. Contingency tables were used to look for associations between subclinical infections and clinical malaria episodes, between cases in houses and individuals in those houses, and between spending the night in the forest or away from the village and both clinical episodes and subclinical infections. Odds ratios were calculated for binary variables and Chi- squared or Fisher’s exact tests were used where appropriate (for variables with multiple levels).

### 2.5 Exploratory spatial analysis

Counts of clinical episodes, subclinical infections and population numbers were aggregated at the individual house level for spatial analysis. Clinical episodes were aggregated into three-month periods matching the timing of the cross sectional surveys. Scan statistics were then used to investigate the timing, location and sizes of potential clusters of either clinical episodes or uPCR detected subclinical infections. Discrete Poisson space-time models were used to estimate statistically significant clusters for each of the villages [19]. Clusters that were statistically significant denote times and areas within study villages in which the relative risk of either a clinical episode or a subclinical infection were higher than expected by chance alone. The scan statistics were run on both clinical episodes and subclinical infections, for both *P. vivax* and *P. falciparum*, and for each of the villages independently (a total of 12 tests). Scan statistics were run using SatScan software (https://www.satscan.org).

Global Moran’s *I* statistics and Local Indicators of Spatial Autocorrelation (LISA) statistics were then used as verification for SatScan results. When clusters were identified using the scan statistics, a Moran’s *I* test was used on the same village and time period to look for concordance between the two approaches. The Moran’s *I* statistic provides an indication of spatial clustering, including the absence or presence of clustering and a measure of clustering magnitude, but it does not indicate the location of clustering when it exists. LISA statistics were therefore used to identify clusters of houses with high numbers of infections. House population sizes were used to standardize infection and clinical episodes counts in houses for both the Moran’s *I* and the LISA statistics. Moran’s *I* and LISA statistics were calculated using ArcMap 10.5.

### 2.6 Logistic regression

Logistic regression was used to calculate model adjusted odds ratios for predictor variables with regard to clinical episodes and uPCR detected subclinical infections of both *P. falciparum* and *P. vivax* (a total of 4 regressions). All regressions, bivariate and univariate analyses were done using R CRAN (http://www.R-project.org/).

### 2.7 Ethics approval

This study was approved by the Oxford Tropical Research Ethics Committee (1015–13) and the Cambodian National Ethics Committee for Health Research (0029 NECHR).

## 3 Results

A total of 1,766 villagers were recorded in the demographic surveillance system, of which 1,520 responded to interviews regarding bednet use, travel or forest dwelling behavior, and 1,593 participated in the cross-sectional surveys using uPCR.

Of the original 1766 individuals recorded in the baseline census, 82% (1447/1,766) participated in the screening at M0 and 33% (575/1,766) participated in all five of the full village surveys (at M0, M3, M6, M9 and M11). Migration in and out of the village influenced the proportion of this cohort that participated. For example, 95 of the 1,447 who participated in M0 did not participate in M3 but did participate in M6 and 49 out of those 95 who missed M3 continued with surveys from M6 onward. The median number of full village surveys in which cohort members participated was 4 (mean 3.14; IQR: 1–5).

During the study period 21 participants (21/1,766; 1.2%) were diagnosed with clinical *P. falciparum* episodes and 81 participants (81/1,766; 4.6%) were diagnosed with clinical *P. vivax* episodes. Of those who participated in the blood screening, 3.3% (53/1,593) were found to carry subclinical *P. falciparum* infections and 8.6% (137/1,593) were found to carry subclinical *P. vivax* infections, and 19% (308/1,593) had *Plasmodium* infections that could not be differentiated at the species level because of low parasite densities. Most clinical episodes (53/81 for *P. vivax* and 17/21 for *P. falciparum*) occurred in one study village (PDB) (Table 1).

**Table 1:** clinical episodes, subclinical infections, reported forest visits and travel history by study village.

	clinical		subclinical		forest visits	travel history	total participants
village	*P. vivax*	*P. falciparum*	*P. vivax*	*P. falciparum*			
KL	23	3	58	24	35	256	657
OK	5	1	16	4	45	126	359
PDB	53	17	63	25	51	222	750
	81	21	137	53	131	604	1766

During the travel surveys 39.7% (604/1,520) of participants reported having spent the night away from the village and 8.6% (131/1,520) reported having spent the night in a forest. Bednet use during the night previous to the questioning was reported by 99% (1,516/1,520) of interviewed participants.

Both the prevalence of uPCR detected malaria and the incidence of clinical cases decreased throughout the study period, during which a well-functioning village malaria worker system was maintained. No bednet distributions were undertaken during the study period.

As previously noted, subclinical *P. vivax* infections in study participants were repeatedly detected through successive uPCR screenings, suggesting relatively long-term chronic infections up to several months [7]. Some of these *P. vivax* carriers also presented with clinical episodes during the study period and in between screenings in which they were detected with subclinical infections through uPCR. For example, in M1 (July 2014) there were 25 clinical *P. vivax* episodes of which five had submicroscopic *P. vivax* infections at M0 (June 2014) and seven at M3 (September 2014; two at both M0 and M3). During M4 (October 2014) there were nine clinical *P. vivax* episodes, with one patient having an uPCR detected subclinical *P. vivax* infection at the M3 screening (September 2014) and four at the M6 screening (December 2014; one participant carrying *P. vivax* parasites at both M3 and M6).

Several individuals experienced repeated clinical episodes of both *P. falciparum* and *P. vivax* during the 19 month study period (Table 2). Five villagers experienced repeated *P. falciparum* episodes, with a median (IQR) time between episodes of 29 days (28–37); while 29 villagers experienced repeated *P. vivax* episodes with a median (IQR) time between episodes of 74 (54.5–109) days. There were also repeated episodes of symptomatic malaria episodes within individual houses. Three houses had multiple episodes of *P. falciparum* malaria, with a median (range) time between episodes of 32 (2.5–222) days while 17 houses had repeated episodes of *P. vivax* malaria with a median (IQR) time between episode of 46.5 (23.5–124) days.

**Table 2:** Timing (in days) between repeated clinical episodes of *P. falciparum* and *P. vivax*, within houses and within individuals. Some houses and individuals experienced more than one repeated episode. “Count” therefore indicates the number of repeated clinical cases (episodes occurring after the initial episode within a house or individual) and “Individuals represented” indicates the number of either houses or individuals in which there were repeated episodes.

Species	Level	Min	Q1	Median	Mean	Q3	Max	Count	Individuals represented
*P. falciparum*	house	0.0	2.5	32.0	124.9	222.0	393.0	7	3
*P. vivax*	house	2.0	23.5	46.5	113.3	124.0	621.0	32	17
*P. falciparum*	individual	20.0	28.0	29.0	95.8	37.0	365.0	5	5
*P. vivax*	individual	13.0	54.5	74.0	91.2	109.0	365.0	55	29

Two-by-two contingency tables indicated associations between subclinical infections and clinical malaria episodes. Participants with a subclinical *P. falciparum* infection had 13 times the odds (unadjusted odds ratio (UOR):13.0; CI: 4.8–34.9) of having a clinical *P. falciparum* malaria episode whereas participants with subclinical *P. vivax* infections had over 18 times the odds (UOR: 18.1; CI: 11.2–29.5) of also having a clinical *P. vivax* malaria episode during the 19-month study period.

Individuals who lived in a house with another person who had a clinical *P. falciparum* malaria episode had approximately 15 times higher odds (UOR: 15.2; CI: 6.2–37.1) of having a clinical *P. falciparum* malaria episode. Participants who had a house member with a clinical *P. vivax* malaria had three times higher odds (UOR: 2.8; CI: 1.8–4.6) of having a clinical *P. vivax* malaria episode. The UOR for an individual with an episode of *P. falciparum* who lived in a house with someone who had a subclinical *P. falciparum* infection was 7.3 (CI: 3.0–17.3). The UOR for an individual with a clinical *P. vivax* malaria episode given that another house member had a subclinical *P. vivax* infection was 2.1 (CI: 1.3–3.3).

The results indicated a strong, positive association between having travelled to forested areas and being detected with subclinical *P. falciparum* and *P. vivax* (uPCR *P. falciparum* UOR: 4.1; CI: 2.1–8.0; uPCR *P. vivax* UOR: 3.40; CI: 2.2–5.4).

We did not find a statistically significant association between clinical or subclinical infection and a history of travel or between clinical malaria and having spent the night in the forest. Bednet usage was uniformly high (99% reported using bednets) and was not further analysed.

### 3.1 Exploratory spatial data analysis

**Fig. 3:**
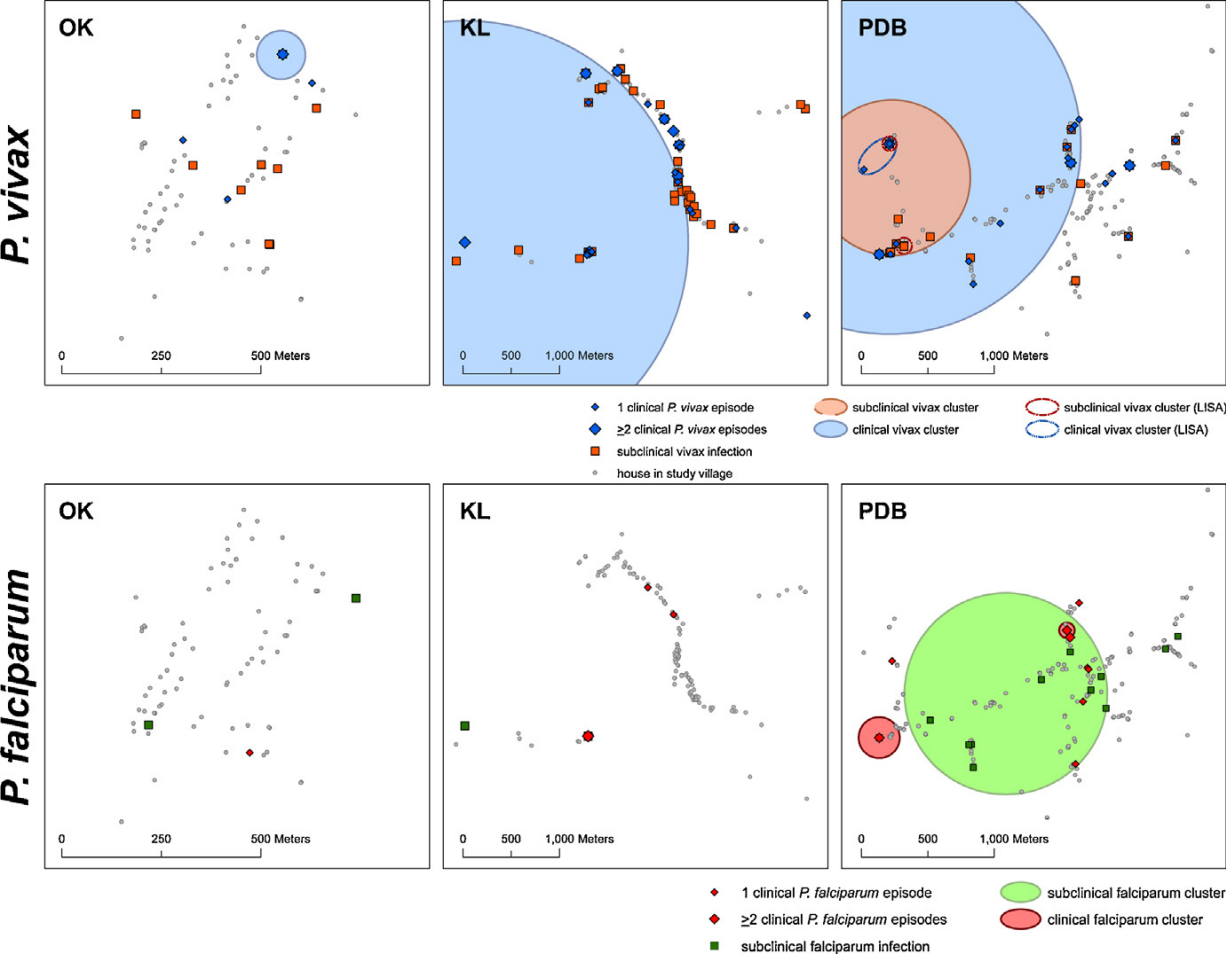
map indicating location of clinical episodes, subclinical infections and clusters for both *P. vivax* (top row) and *P. falciparum* (bottom row). Clusters for clinical *P. vivax* episodes (blue circles) occurred during M9 for villages OK and KL and during M3 for village PDB. The cluster for subclinical *P. vivax* (orange circle) occurred in village PDB from M3 to M6. The clusters for clinical *P. falciparum* episodes (red circles) occurred in village PDB during M0–M5. The cluster for subclinical *P. falciparum* (green circle) occurred in village PDB during M0.

Most clinical *P. falciparum* episodes, clinical *P. vivax* episodes, subclinical *P. falciparum* and subclinical *P. vivax* infections (both total counts and proportion positive) occurred within one study village: PDB (Fig. 3). Conversely, the proportion of villagers reporting having travelled or spent the night in the forest was higher in other villages.

There was no statistically significant difference between participants with and without clinical *falciparum* episodes with regard to living near another house with a clinical *falciparum* infection. Participants with a clinical *vivax* episode lived closer to other houses with clinical *vivax* episodes (a median distance of 48 meters compared to 103 meters for participants without a clinical *vivax* episode; Wilcox rank sum test p-value 0.0062).

The scan statistics indicated clusters of clinical *P. vivax* episodes in each of the study villages (Fig. 3). The relative risk of infection within these clusters was significantly higher in comparison to the population outside of the cluster. Single clusters were found in village KL and OK during M6–M8 (December 2013–February 2014) and in village PDB during months M0–M2 (June–August 2013). The clusters of clinical *P. vivax* in KL and OK were not confirmed with the Moran’s *I* statistics. The cluster in village OK consisted of a single household only, with two *P. vivax* clinical episodes occurring among five house members. There were two statistically significant clusters of clinical *P. falciparum* in village PDB during M0 through M5 (June–November 2013), one of which consisted of a single household with four cases among eight inhabitants. Neither cluster of *P. falciparum* was detected using the Moran’s *I* statistics. PDB was also the only village to have clusters of subclinical *Plasmodium* infections, with a single cluster of subclinical *P. falciparum* in M0 and a single cluster of subclinical *P. vivax* (in M3 and M6 (September and December 2013) combined). The cluster of subclinical *P. falciparum* was not detected by the Moran’s *I* statistic but the cluster of subclinical *P. vivax* infections was. The cluster of subclinical *P. vivax infections* (M3 and M6 (September and December 2013)) overlapped but was smaller than the cluster of clinical *P. vivax* (M0–M2 (June–August 2013)), detected by scan statistics, Moran’s *I* and LISA statistics. The cluster of subclinical *P. falciparum* (M0 (June 2013)) overlapped with one of the clinical *P. falciparum* clusters (M0–M5 (June–November 2013)). The single house cluster of *P. falciparum* in the village PDB occurred within the spatial clusters with clinical *P. vivax* episodes and uPCR detected subclinical *P. vivax* clusters (Fig. 3).

### 3.2 Logistic regressions and adjusted odds ratios

#### 3.2.1 Clinical *P. falciparum* and *P. vivax* malaria episodes

The strongest predictor for clinical *P. falciparum* malaria episodes was living in a house with another person that had a clinical *P. falciparum* malaria episode. Individuals who lived in a house with someone who had a clinical *P. falciparum* malaria had 7 times the odds (adjusted odds ratio (AOR): 7.15; CI: 2.4–20. 5) of having a clinical *P. falciparum* episode (Table 3). Having a subclinical *P. falciparum* infection (AOR: 5.9; CI: 1.5–19.8) and living in a house with someone else who had a subclinical *P. falciparum* infection (AOR: 4.2; CI: 1.5–12.1) were also strong predictors of clinical *P. falciparum* malaria during the 19 month study period. Participants who lived in village PDB had 6 times the odds (AOR: 6.10; CI: 1.5–42.5) of having a *P. falciparum* episode compared to participants living in village KL. There were no clear sex or age patterns in clinical *P. falciparum* episodes.

**Table 3:** Logistic regression for odds of having a clinical *P. falciparum* episode.

covariate	total	Pf episodes	UOR	UOR CI	AOR	AOR CI	p value
0–9 years old	462	3	0.47	(0.14–1.59)	comparison group		
10–45 years old	1025	16	2.33	(0.85–6.40)	2.01	(0.61–9.14)	0.2960
>45 years old	279	2	0.56	(0.13–2.41)	0.32	(0.01–3.29)	0.3947
female	865	7			comparison group		
male	901	14	1.93	(0.78–4.82)	1.52	(0.55–4.48)	0.4227
did not report staying in forest	1389	17			comparison group		
reported staying in forest	131	3	1.89	(0.55–6.54)	1.67	(0.33–6.20)	0.4806
did not report traveling	916	12			comparison group		
reported traveling	604	8	1.01	(0.41–2.49)	1.74	(0.62–4.77)	0.2804
no subclinical Pf	1540	15			comparison group		
subclinical Pf	53	6	12.98	(4.82–34.94)	5.93	(1.53–19.77)	0.0072
no house member with Pf episode	1675	12			comparison group		
house member with Pf episode	91	9	15.21	(6.23–37.12)	7.15	(2.40–20.49)	0.0003
no house member with subclinical Pf	1525	10			comparison group		
house member with subclinical Pf	241	11	7.25	(3.04–17.25)	4.23	(1.46–12.11)	0.0056
distance to nearest house with Pf episode					1.00	(0.99–1.01)	0.9165
Village KL	657	3	0.28	(0.08–0.95)	comparison group		
Village OK	359	1	0.19	(0.03–1.45)	1.46	(0.07–16.38)	0.7621
Village PDB	750	17	5.87	(1.97–17.51)	6.10	(1.47–42.50)	0.0276
number of surveys attended					0.86	(0.61–1.24)	0.4123

*Total of 1,511 participants with full data included in the regression.

Pf indicates *P. falciparum*.

The strongest predictor for having a clinical *P. vivax* episode was having a subclinical *P. vivax* infection. Participants who were found to have subclinical infections had over 13 times the odds (AOR: 13.3; CI: 7.7–23.0) of having a clinical *P. vivax* episode, after controlling for other potentially important factors (Table 4). *P. vivax* malaria episodes also tended to cluster in the 10–45 age group (AOR: 3.4; CI: 1.6–8.1) and in males (AOR: 1.8; CI: 1.0–3.2). As with clinical *P. falciparum* episodes, clinical *P. vivax* episodes were predominantly clustered in village PDB. Participants who were from PDB had an AOR of 2.1 (CI: 1.2–3.9) when compared to village KL.

**Table 4:** Logistic regression for odds of having a clinical *P. vivax* episode.

covariate	total	Pv episodes	UOR	UOR CI	AOR	AOR CI	p value
0–9 years old	462	8	0.3	(0.14–0.62)	comparison group		
10–45 years old	1025	67	3.63	(2.03–6.51)	3.39	(1.62–8.06)	0.0026
>45 years old	279	6	0.41	(0.18–0.96)	1.03	(0.32–3.19)	0.9555
female	865	25			comparison group		
male	901	56	2.23	(1.38–3.60)	1.80	(1.04–3.17)	0.0384
did not report staying in forest	1389	70			comparison group		
reported staying in forest	131	10	1.56	(0.78–3.10)	0.63	(0.27–1.38)	0.2694
did not report traveling	916	49			comparison group		
reported traveling	604	31	0.96	(0.60–1.52)	1.20	(0.70–2.03)	0.50347
no subclinical Pv	1456	37			comparison group		
subclinical Pv	137	44	18.14	(11.17–29.47)	13.25	(7.71–22.98)	<0.0001
no house member with Pv episode	1460	52			comparison group		
house member with Pv episode	306	29	2.83	(1.77–4.55)	1.69	(0.72–2.46)	0.1095
no house member with subclinical Pv	1261	45			comparison group		
house member with subclinical Pv	505	36	2.07	(1.32–3.26)	1.34	(0.72–2.46)	0.3532
distance to nearest house with Pv episode					1.00	(0.99–1.00)	0.3483
Village KL	657	23	0.66	(0.40–1.08)	comparison group		
Village OK	359	5	0.25	(0.10–0.62)	0.50	(0.16–1.36)	0.2064
Village PDB	750	53	2.68	(1.68–4.28)	2.11	(1.18–3.86)	0.0131
number of surveys attended					1.08	(0.89–1.33)	0.44726

*Total of 1,511 participants with full data included in the regression.

Pv indicates *P. vivax*.

Distance to the nearest house with a clinical infection showed no statistical significance for either clinical *P. falciparum* or *P. vivax* episodes after controlling for covariates.

#### 3.2.2 Subclinical *P. falciparum* and *P. vivax* infections

The largest predictors of having a subclinical *P. falciparum* infection was self-reported traveling to the forest (Adjusted Odds Ratio (AOR) = 3.5; CI: 1.6–7.1) or having a clinical *P. falciparum* malaria episode during the study period (AOR: 5.7; CI: 1.4–19.6) (Table 5).

**Table 5:** Logistic regression for odds of having a subclinical *P. falciparum* infection.

covariate	total	Pf infections	UOR	UOR CI	AOR	AOR CI	p value
0–9 years old	430	10	0.62	(0.31–1.25)	comparison group		
10–45 years old	907	31	1.07	(0.61–1.86)	1.18	(0.57–2.64)	0.6716
>45 years old	256	12	1.55	(0.81–3.00)	1.75	(0.68–4.49)	0.2378
female	782	19			comparison group		
male	811	34	1.76	(0.99–3.11)	1.71	(0.91–3.32)	0.1011
did not report staying in forest	1385	36			comparison group		
reported staying in forest	131	13	4.13	(2.13–8.00)	3.49	(1.63–7.14)	0.0008
did not report traveling	913	32			comparison group		
reported traveling	603	17	0.8	(0.44–1.45)	0.85	(0.44–1.59)	0.6178
no Pf episode	1572	47			comparison group		
Pf episode	21	6	12.98	(4.82–34.94)	5.70	(1.44–19.59)	0.0083
no house member with Pf episode	1505	44			comparison group		
house member with Pf episode	88	9	3.78	(1.78–8.02)	2.01	(0.72–4.87)	0.1490
no house member with subclinical Pf	1367	34			comparison group		
house member with subclinical Pf	226	19	3.6	(2.01–6.43)	1.93	(0.94–3.78)	0.0628
distance to nearest house with Pf episode					1.00	(1.00–1.01)	0.4048
Village KL	588	24	1.43	(0.83–2.48)	comparison group		
Village OK	324	4	0.31	(0.11–0.87)	0.29	(0.08–0.81)	0.0304
Village PDB	681	25	1.2	(0.70–2.08)	0.81	(0.42–1.58)	0.5383
number of surveys attended					1.16	(0.93–1.47)	0.2135

*Total of 1,511 participants with full data included in the regression.

Several factors were statistically significant predictors of subclinical *P. vivax* infection. The strongest predictor was having a clinical *P. vivax* malaria episode during the study period (AOR: 14.1; CI: 8.2–24.5) (Table 6). Individuals with subclinical *P. vivax* infections tended to be male (AOR: 2.4; CI: 1.6–3.8). The risk of being detected with subclinical *P. vivax* infections increased with age, participants above age nine having 2.6 times the odds (CI: 1.5–4.9) of carrying *P. vivax* infections and those above age 45 with 2.9 times the odds (CI: 1.4–5.9) of being detected with subclinical *P. vivax* infection (both in comparison to the 0–9 age group). Staying overnight in a forested area also had a strong association with subclinical *P. vivax* infections (AOR = 2.4; CI: 1.4–4.1). Cohort members who participated in more surveys also had a higher odds of being detected with a subclinical P. vivax infection (AOR = 1.5; CI: 1.3–1.8).

**Table 6:** Logistic regression for odds of having a subclinical *P. vivax* infection.

covariate	total	Pv infections	UOR	UOR CI	AOR	AOR CI	p value
0–9	430	16	0.33	(0.20–0.57)	comparison group		
10–45	907	98	2.01	(1.37–2.95)	2.62	(1.49–4.87)	0.0014
46 Plus	256	23	1.06	(0.66–1.69)	2.85	(1.42–5.86)	0.0036
female	782	39			comparison group		
male	811	98	2.62	(1.78–3.85)	2.43	(1.58–3.79)	0.0001
did not report staying in forest	1385	107			comparison group		
reported staying in forest	131	29	3.4	(2.15–5.36)	2.42	(1.40–4.10)	0.0013
did not report traveling	913	78			comparison group		
reported traveling	603	58	1.14	(0.80–1.63)	1.12	(0.74–1.69)	0.58496
no Pv episode	1512	93			comparison group		
Pv episode	81	44	18.14	(11.17–29.47)	14.08	(8.17–24.50)	<0.0001
no house member with Pv episode	1311	100			comparison group		
house member with Pv episode	282	37	1.83	(1.22–2.73)	1.08	(0.62–1.84)	0.7723
no house member with subclinical Pv	1121	78			comparison group		
house member with subclinical Pv	472	59	1.91	(1.34–2.73)	1.54	(0.97–2.45)	0.0673
distance to nearest house with Pv episode					1.00	(1.00–1.01)	0.1645
Village KL	588	58	1.28	(0.90–1.83)	comparison group		
Village OK	324	16	0.49	(0.29–0.84)	0.37	(0.19–0.69)	0.0023
Village PDB	681	63	1.15	(0.81–1.64)	0.61	(0.39–0.96)	0.0327
number of surveys attended					1.50	(1.27–1.80)	<0.0001

*Total of 1,511 participants with full data included in the regression.

## 4 Discussion

This study found clustering of both clinical cases subclinical parasite carriage at multiple scales within the villages. While the study villages are within three to four kilometers from each other, the majority of clinical malaria episodes and subclinical infections occurred in one village (PDB), which was home to 67% (70/102) of all malaria episodes and 46% (88/190) of all subclinical infections.

This study found cases clustering at levels below the village at specific times during the 19 month study period. Clustering of clinical *P. vivax* episodes was most common, occurring in all three study villages. Clustering of clinical *falciparum* episodes occurred only in one village, and was confined to small geographical areas (one cluster consisted of a single house while the other consisted of four neighbouring houses). Clustering of subclinical infections only occurred in one village (PDB), with the subclinical *vivax* cluster overlapping with a cluster of clinical *vivax* malaria cases that occurred at the beginning of the study.

These findings have implications with regard to strategies for elimination and control efforts, especially those that use spatial targeting. If an approach of reactive case detection screening surrounding households with a sensitive diagnostic had been used it may have occasionally resulted in the detection of other cases within the same index house. This study found that clinical *P. falciparum* cases and subclinical *P. falciparum* carriers tend to occur within the same house during the study period. However, these within-house clusters account for a small proportion of all cases. For example, 43% (9/21) of clinical *P. falciparum* episodes were in participants who lived in a household with at least one other villager who had a subclinical *P. falciparum* infection during the study period. Under half of all participants who had clinical *P. vivax* episodes (36/81) shared a house with another villager who had a subclinical *P. vivax* infection. Interventions that targeted index households would therefore have been missed over half of all subclinical infections, even if diagnoses had been made using a highly sensitive approach and if the timing of those subclinical infections had been properly predicted. Clinical episodes of *P. falciparum* malaria occasionally clustered within individual houses (Table 2, Table 3). In four out of the seven houses with multiple *P. falciparum* episodes during the study period, the timing between episodes was within a month of each other (Table 2). One house experienced double episodes (with two house members having clinical *P. falciparum* episodes on the same day) at two different points in time (once in October 2013 and again in November 2014). Though there was statistically significant clustering of *P. falciparum* within houses in the study period, household screening and treatment within a month of the index case would have detected only 20% of the clinical *P. falciparum* episodes (four out of a total of 21).

Clinical episodes did not consistently cluster in groups of houses during the study period. The logistic regression models indicated that the distance to the nearest neighbor was not a statistically significant predictor of an individual having a clinical infection, for either *P. falciparum* or *P. vivax*. While the scan statistics did indicate spatio-temporal clustering, the clustering was not consistent across time and less than half (34/81; 42% *P. vivax* and 9/21; 43% *P. falciparum*) of all clinical episodes were recorded within these detectable clusters. Follow up verification of the scan statistics through the use of Moran’s *I* statistics could only verify 2 out of the total of 6 statistically significant clusters (referring to the partially overlapping clusters of subclinical *P. vivax* infections and clinical *P. vivax* episodes in village PDB). This further supports the limitations of identifying and predicting clustering of infections within villages in this setting. Conversely, an intervention treating presumptively one entire village (PDB) or using a highly sensitive detection and treatment approach could have accounted for most (but not all) of the cases in the three villages (Table 1).

Participants who reported spending the night in a nearby forest were more likely to have subclinical infections (both *P. vivax* and *P. falciparum*) during the study period when compared to participants who did not report a forest visit. This finding, in combination with sporadic and transient clustering of cases within the villages, suggests that transmission also occurred outside of the village, and warrants specific targeting of forest goers.

These data suggest that a considerable proportion of individuals in villages may have chronic, low-density *P. vivax* infections [6, 7] and that there is a strong association between subclinical infections and malaria episodes. These data also suggest that subclinical infections may eventually become clinical episodes. Some participants were found subclinically infected by *P. vivax* after having presented with a clinical episode. Also in village PDB, the cluster of subclinical *P. vivax* infections occurred after the cluster of clinical *P. vivax* malaria episodes. Since curative doses of primaquine were not used in the villages at the time of the study, it is plausible that these were relapse infections rather than reinfections.

In the presence of viable mosquito vectors, individuals with subclinical infections over long periods of time may be important contributors to ongoing transmission in this region. If *P. vivax* is targeted for elimination it will be important to address chronic, subclinical infections. Our data suggest that spatial targeting at levels smaller than the village may not adequately address this potential reservoir.

Finally, both clinical and subclinical *P. falciparum* infections in these study villages decreased to almost zero in all study villages despite the absence of any additional targeted interventions other than sustaining a well-functioning village based malaria worker system providing early diagnosis and treatment. *P. vivax* infections also decreased but the decrease was less pronounced than that of *P. falciparum* infections. Village health workers are relatively easy to implement, cost efficient and can have a profound effect on *P. falciparum* incidence [17, 20]. Other factors may have led to the decline in *P. falciparum* in these study villages, but they occurred in the absence of expensive and labour heavy approaches (e.g. screen and treat approaches). In order to see a similar effect on *P. vivax* incidence it will be necessary to target liver stage parasites with radical treatment, as well as subclinical, chronic infections.

There are several limitations to this study. It is possible that some villagers with febrile illness attended health facilities outside of the village clinics and these cases would have been missed in this analysis. Some of the uPCR detected infections could not be identified at the species level. Low numbers of *P. falciparum* infections, especially clinical *P. falciparum* cases, made sophisticated analyses difficult and led to wide confidence intervals around model predictions. However, all significant covariates in the statistical models were also significant in univariate analyses and while large odds ratios may be over-predictions they indicate strong correlations. Participation in surveys was associated with increased odds of an individual being detected with at least one subclinical *P. vivax* infection. The numbers of people detected with subclinical *P. vivax* infections here may therefore be an underestimate.

Another limitation to this work is reporting bias in bednet use and travel interviews. Participants may have forgotten important travel histories or may have been hesitant to admit travelling to specific areas (for example, to forests for illegal logging). This type of bias is likely to have a stronger influence with regard to false negatives than to false positives. Positive associations are likely to remain despite this bias whereas a finding of no correlation may be the result of under-reporting. Finally, the variable for having spent the night in the forest within the previous two weeks may be prone to bias in that no specific definition of “forest” was given by either interviewer or interviewee. It is therefore possible that different participants have different understandings of what constitutes a forest. Furthermore, the question regarding forest stays and travelling did not specify the amount of time spent either in the forest or away from the village. If forests are an important source of *Plasmodium* infections then it would be valuable to know if longer exposure to forests is associated with higher risk of infection. Future research will aim to address this question.

## 5 Conclusion

These data contribute to a growing body of literature that illustrate the inherent difficulties of predicting spatio-temporal clustering of malaria at scales above the household level and below the village level. While helpful for directly (human-to-human) transmitted diseases with relatively short incubation periods (e.g. smallpox), predicting the transmission of vector borne diseases is more challenging. This is likely the result of complex transmission patterns and frustrates interventions that are based on prediction of space time occurrence of malaria at these scales (such as reactive case detection). Finally, elimination of *P. vivax* will require curative treatment of hypnozoites, with subclinical infections potentially playing an important role in *P. vivax* transmission.

## Declarations

### Author contribution statement

Daniel Parker, Richard Maude: Analyzed and interpreted the data; Wrote the paper.

Rupam Tripura, Thomas Peto: Conceived and designed the experiments; Performed the experiments; Analyzed and interpreted the data; Wrote the paper.

Mallika Imwong: Analyzed and interpreted the data; Contributed reagents, materials, analysis tools or data, Wrote the paper.

Pasathorn Sirithiranont, Jeremy Chalk, Chea Nguon: Performed the experiments, Wrote the paper.

Lorenz von Seidlein, Nicholas White, Arjen Dondorp: Conceived and designed the experiments; Wrote the paper.

### Competing interest statement

The authors declare no conflict of interest.

### Funding statement

This work was supported by the Bill & Melinda Gates Foundation and the Wellcome Trust of Great Britain.

### Additional information

No additional information is available for this paper.
